# ADAPTING TELEHEALTH EVIDENCED BASED PROTOCOLS FOR VETERANS WITH MOOD AND NEUROCOGNITIVE DISORDERS: A CASE STUDY

**DOI:** 10.1093/geroni/igad104.3265

**Published:** 2023-12-21

**Authors:** Charissa Hosseini

**Affiliations:** Veterans Health Administration, Alameda, California, United States

## Abstract

Aging Veterans with mood and neurocognitive disorders typically have a complex medical and psychiatric presentations leading many providers to exclude them from treatment, expressing that psychotherapy is not appropriate. This form of exclusionary care may decrease help seeking behavior and amplify existing psychological symptoms to an already stigmatized and isolated population. The following case study, outlines a 24-session approach to integrating evidenced based protocols such as Cognitive Behavioral Therapy (CBT) and Narrative Life Review Therapy via telehealth. The presentation will discuss cultural considerations to take when working with Aging Veterans that have co-occurring mood and cognitive difficulties as well as reflect upon the successes and challenges of providing telehealth with this population.

